# Risk factors for laminitis and nonsurvival in acute colitis: Retrospective study of 85 hospitalized horses (2011‐2019)

**DOI:** 10.1111/jvim.16147

**Published:** 2021-05-03

**Authors:** Daniela Luethy, Rose Feldman, Darko Stefanovski, Maia R. Aitken

**Affiliations:** ^1^ Department of Large Animal Clinical Sciences University of Florida College of Veterinary Medicine Gainesville Florida USA; ^2^ Department of Clinical Studies‐New Bolton Center, School of Veterinary Medicine University of Pennsylvania Kennett Square Pennsylvania USA

**Keywords:** coronavirus, neorickettsiosis, salmonellosis

## Abstract

**Background:**

Acute colitis is a serious cause of morbidity and death in horses. Recent studies have compared clinical features of coronavirus and salmonellosis, but no study has compared clinical features of enteric salmonellosis, coronavirus, and neorickettsiosis.

**Hypothesis/Objectives:**

To identify risk factors for laminitis and nonsurvival to discharge in horses with enteric salmonellosis, coronavirus, or neorickettsiosis.

**Animals:**

Eighty‐five horses hospitalized for acute colitis from 2011 to 2019.

**Methods:**

Retrospective case series. Medical record review (2011‐2019) of adult (≥2 years) horses with colitis. Primary outcomes were laminitis and survival to discharge. Multivariable logistic regression was performed to assess association between variables and the development of laminitis. Stepwise Cox regression was performed to assess association between variables and survival.

**Results:**

Seventeen of 85 (20%) horses developed laminitis during hospitalization. Neorickettsiosis cases (11/26, 42%) were more likely to develop laminitis than coronavirus (0/16, 0%) cases (odds ratio [OR] 24.48; 95% confidence interval [CI]: 1.33‐451.74, *P* = .03). There was no significant difference in laminitis between salmonellosis and neorickettsiosis cases (OR 0.27; 95% CI: 0.07‐1.07, *P* = .06). Admission heart rate (OR 1.08; 95% CI: 1.02‐1.15, *P* = .01), total solids (OR 0.17; 95% CI: 0.06‐0.54, *P* = .003), band neutrophils (OR 1248.47; 95% CI: 6.62‐235 540, *P* = .008), and bicarbonate concentration (OR 0.68; 95% CI: 0.5‐0.92, *P* = .01) were predictive of development of laminitis during hospitalization. Sixty‐three of 85 (74%) horses survived to discharge: 16/16 (100%) coronavirus cases, 17/26 (65%) neorickettsiosis cases, 14/20 (70%) salmonellosis cases, and 16/23 (70%) unknown cases. Packed cell volume (hazard ratio [HR] 1.17; 95% CI: 1.09‐1.26, *P* < .001), L‐lactate concentration (HR 1.33; 95% CI: 1.14‐1.55, *P* < .001), and development of laminitis (HR 7.07; 95% CI: 1.67‐29.95, *P* = .008) were retained in the final multivariable model for prediction of nonsurvival to discharge.

**Conclusion and Clinical Importance:**

Nonsurvival and laminitis rates were high, likely related to the presence of neorickettsiosis in the region.

**Abbreviations**
HR: hazard ratio
OR: odds ratio

## 1 INTRODUCTION

Acute colitis or typhlocolitis is a serious cause of morbidity and death in horses.^1^ Horses with acute colitis often present with diarrhea, fever, colic, and signs of endotoxemia. Common causes of acute infectious colitis in adult horses include *Salmonella enterica*, *Neorickettsia risticii* (Potomac Horse Fever), *Clostridium difficile* and *perfringens*, and equine coronavirus.^1^ Equine coronavirus is a recently emerging enteric pathogen of horses with a wide variety of clinical presentations, from acute severe diarrhea to fever and colic to lymphopenia.^2^, ^3^, ^4^, ^5^, ^6^, ^7^

A recent study of enteric coronavirus and salmonellosis in 42 horses reported a survival rate of 98%, and 1/42 (2.4%) horses in that study developed laminitis.^5^ This, however, is in stark contrast to the clinical impression for survival and laminitis in regions of the country where neorickettsiosis is a common cause of colitis. For example, a study of enteric neorickettsiosis in 44 horses reported a 73% survival rate and 16/44 horses (36%) developed laminitis.^8^ Risk factors associated with nonsurvival in that study are reflective of severity of disease and included hemoconcentration, hypochloremia, hyponatremia, azotemia, and duration of hospitalization.^8^ The severity of neorickettsiosis varies widely between geographic regions.^9^

Despite the various etiologic agents associated with colitis and the variability of clinical signs associated with enteric coronavirus, horses presenting for acute colitis can have indistinguishable clinical signs and clinicopathologic data. To date, no study has compared the clinical features of enteric salmonellosis, coronavirus, and neorickettsiosis within a single hospitalized population to determine risk factors for laminitis and nonsurvival to discharge. Therefore, our objectives were to (a) compare the clinical features of enteric salmonellosis, enteric coronavirus, and enteric neorickettsiosis and (b) identify risk factors for the development of laminitis as well as nonsurvival to discharge in horses with colitis caused by *Salmonella*, coronavirus, and *N risticii*.

## 2 MATERIALS AND METHODS

Cases were identified by an electronic medical record database search from 2011 to 2019 for adult (≥2 years) horses treated at the George D Widener Hospital for Large Animals at the University of Pennsylvania's New Bolton Center with a presenting complaint or final diagnosis of colitis. Horses were included in the study if diagnosed with enteric salmonellosis, enteric coronavirus, or enteric neorickettsiosis based on fecal polymerase chain reaction (PCR) testing, or a colitis of open etiology. Horses were excluded if fecal PCR testing was not performed for all 3 pathogens, if diagnosis was presumptive, or if colitis developed after exploratory celiotomy. Information recorded for each patient included: signalment, admission date, admission season (Spring, Summer, Fall, Winter), history, presenting complaint, physical examination findings, clinicopathologic data, fecal testing results, treatment, adverse events, development of laminitis, total hospitalization days and cost, and necropsy results (where applicable). In our hospital, a negative *Salmonella* status requires 3 consecutive negative fecal PCR results. Therefore, horses were classified as enteric salmonellosis if the horse had 1 or more positive fecal *Salmonella* PCR results and 1 negative result each for coronavirus and *N risticii* PCR. Horses were classified as enteric coronavirus if the horse had 1 positive fecal coronavirus PCR result, 1 negative result for *N risticii*, and 3 negative results for *Salmonella*. Horses were classified as enteric neorickettsiosis if the horse had 1 positive fecal *N risticii* PCR result, 1 negative result for coronavirus, and 3 negative results for *Salmonella*. Horses were classified as unknown if the horse had 3 negative fecal *Salmonella* PCRs, 1 negative coronavirus PCR, and 1 negative *N risticii* PCR. If a horse did not survive long enough to obtain 3 consecutive negative fecal *Salmonella* PCR results, necropsy diagnosis was used for final diagnosis. *C perfringens* and *difficile* enterocolitis was initially included in the study design; however, only a single horse was identified to be *C difficile* positive, and therefore clostridial enterocolitis was not included in results and this single horse was excluded from analysis.

Treatments administered in hospital were dependent on admitting clinician preference. In general, this consisted of IV administration of balanced isotonic crystalloid fluid, flunixin meglumine, polymyxin B, intragastric administration of di‐tri‐octahedral smectite (Biosponge powder, Platinum Performance Inc, Buellton, California), antibiotics (if indicated as discussed below), and distal limb cryotherapy (Jack's Boots, Jack's Inc, Washington, Ohio). Sixty of 85 horses received antibiotics during hospitalization. Because of the presence of neorickettsiosis in the region, horses admitted with signs of colitis in summer months (June‐September) routinely received oxytetracycline IV initiated at admission. All horses with a final diagnosis of neorickettsiosis received oxytetracycline IV. In nonsummer months, antimicrobial administration was limited to horses with a neutrophil count of <1000/μL or concern for an additional site of infection (pneumonia, thrombophlebitis). Fifty percent of horses with a diagnosis other than neorickettsiosis received antimicrobials.

A diagnosis of laminitis was based on clinical examination by the supervising clinician and was defined as an Obel grade of 1, 2, 3, or 4 with subjectively increased digital pulse pressure, clefting of the coronary band, or both, with or without confirmatory radiographic changes.^10^, ^11^ Specific treatments for laminitis included nonsteroidal anti‐inflammatory drugs, digital cryotherapy, solar support, pentoxifylline, lidocaine continuous rate infusion, opioids (morphine, butorphanol), gabapentin, and in severe cases, coronary band grooving and foot casts. Nonsurvival was defined as death or euthanasia before discharge; horses who survived to discharge were not followed beyond discharge. In horses that were euthanized, reason for euthanasia was poor prognosis associated with their primary condition (colitis) or because of poor prognosis and poor quality of life associated with severe laminitis.

### 2.1 Statistical analysis

Statistical analysis was performed using standard statistical software (Stata 15.1MP, StataCorp, State College, Texas). Data were assessed for normality using the Shapiro‐Wilk test for normality. Normally distributed variables were reported as mean ± SD. Non‐normally distributed variables were reported as median and range. Categorical variables were compared using a Chi‐Square test. For continuous variables, normal data were compared across groups using a 1‐way ANOVA with post hoc Bonferroni correction, whereas non‐normal data were compared across groups using Kruskal‐Wallis. Horses were grouped based on final diagnosis, and coronavirus was set as the reference, with the exception of survival analysis, where neorickettsiosis was set as the referent because no coronavirus cases died. Univariate logistic regression was performed to assess association between variables and the development of laminitis, followed by lasso regression to build significant variables into the final predictive multivariable logistic model. Lasso regression is a novel method for selection in multivariable models. In Lasso regression, independent variables are selected and fit to linear, logistic, probit, and Poisson models, and the results of lasso regression are used for multivariable model selection.^12^ Univariate Cox regression was performed to confirm the association with the outcome of interest (survival), followed by stepwise Cox regression with Breslow method for ties for determination of the final multivariable Cox regression model with factors that were significantly associated with nonsurvival. Because of complete separation of data, Firth logistic regression was then used. All analysis was conducted with 2‐sided tests of hypotheses and *P*‐value of <.05 set as the criterion for statistical significance.

## 3 RESULTS

### 3.1 Animals

Eighty‐six horses met the inclusion criteria. A single horse was identified which was both coronavirus and *Salmonella* PCR positive, and this horse was excluded from further analysis. Therefore, 85 horses were included in the final study. Median age was 8 years (range, 2‐26 years). There were 46 mares, 27 geldings, and 12 stallions. Breeds included Thoroughbred/Thoroughbred cross (n = 32), Quarter Horse/Paint (n = 13), Standardbred (n = 11), Warmblood (n = 10), Draft (n = 6), Pony (n = 5), Morgan (n = 3), Miniature Horse (n = 2), Appaloosa (n = 1), Friesian (n = 1), and Arabian (n = 1). Horses were most commonly admitted in summer (n = 45), followed by spring (n = 17), winter (n = 15), and fall (n = 8). Median number of days of hospitalization was 5.63 days (range, 1‐24 days). Most common presenting complaints included fever (n = 41), diarrhea (n = 34), and colic (n = 25).

### 3.2 Diagnosis

Twenty‐six of 85 (31%) horses were classified as neorickettsiosis, 20/85 (24%) were classified as salmonellosis, 16/85 (19%) were classified as coronavirus, and 23/85 (27%) were classified as unknown.

### 3.3 Clinical findings

Table 1 summarizes clinical findings at admission stratified by final diagnosis. Coronavirus cases were significantly less likely to have diarrhea than other diagnoses (*χ* ^2^ = 15.38; *P* = .002) and significantly more likely to display colic signs than other diagnoses (*χ* ^2^ = 27.91; *P* = .001).

**TABLE 1:** Clinical findings of 85 horses hospitalized for acute colitis, stratified by diagnosis. For continuous data, non‐normal data are listed as median and range with *P* value generated by Kruskal‐Wallis, and normal data are listed as mean ± SD with *P* value generated by 1‐way ANOVA with post hoc Bonferroni for any significant values. For categorical data, *P* value is generated by chi‐square. % represents within‐column frequency

	Neorickettsiosis (n = 26)	Salmonellosis (n = 20)	Coronavirus (n = 16)	Unknown (n = 23)	*P* value
Age (years)	11 (2‐26)	5.5 (2‐15)	6 (2‐24)	8 (2‐20)	.62
Total hospitalization days	4.5 (1‐17)	8 (2‐24)	6 (2‐15)	4 (1‐14)	.25
Total hospital bill ($)	5125 ± 3220	7518 ± 4771	3594 ± 2073	5171 ± 3878	.66
Admission heart rate (bpm)	60 (36‐96)	58 (40‐80)	46 (40‐72)	60 (40‐108)	.66
Admission respiratory rate (brpm)	24 (10‐60)	28 (16‐80)	22 (16‐60)	20 (12‐52)	.49
Admission rectal temperature (°F)	101.1 (98.0‐104.1)	101.5 (98.6‐105.7)	100.8 (99.3‐105.1)	100.3 (97.3‐102.7)	.57
Fecal consistency ‐ diarrhea	17 (65%)	12 (60%)	2 (13%)	15 (65%)	.002
Colic signs during hospitalization	3 (12%)	5 (25%)	13 (81%)	8 (35%)	.001
Laminitis developed in hospital	11 (42%)	3 (15%)	0 (0%)	3 (13%)	.03
Survival to discharge	17 (65%)	14 (70%)	16 (100%)	16 (70%)	Incalculable

Table 2 summarizes clinicopathologic findings at admission stratified by final diagnosis. There were no significant differences between groups for any complete blood count values, or for admission PCV, total solids, or peripheral L‐lactate concentration. There were no significant differences between groups for any chemistry panel values except for plasma sodium concentration: neorickettsiosis cases had significantly lower sodium concentration than coronavirus cases (125.96 ± 5.51 mmol/L vs 131 ± 2.85 mmol/L, *P* = .02), lower sodium concentration than salmonellosis cases (125.96 ± 5.51 mmol/L vs 131.13 ± 4.64 mmol/L, *P* = .02) and lower than unknown cases (125.96 ± 5.51 mmol/L vs 130.86 ± 6.03 mmol/L, *P* = .01).

**TABLE 2:** Clinicopathologic findings of 85 horses hospitalized for acute colitis, divided by diagnosis. Non‐normal data listed as median and range with *P* value generated by Kruskal‐Wallis, normal data listed as mean ± SD with *P* value generated by 1‐way ANOVA with post hoc Bonferroni for any significant values

	Reference ranges	Neorickettsiosis (n=26)	Salmonellosis (n=20)	Coronavirus (n=16)	Unknown (n=23)	*P* value
Packed cell volume (%)	30‐45	50 (28‐65)	41.5 (31‐57)	36.5 (32‐50)	42 (30‐78)	.87
Total solids (g/dL)	6.0‐7.5	6.59 ± 1.52	6.67 ± 1.12	6.65 ± 0.55	6.42 ± 1.17	.9
Lactate (mmol/L)	<2.0	2.65 (0.7‐14.7)	1.6 (0.7‐13.2)	1.3 (0.6‐4.5)	2.45 (1.0‐12)	.56
Blood glucose (mg/dL – glucometer)	72‐114	130.45 (101‐353.3)	128 (50‐173)	124.8 (91.9‐180.4)	130 (55‐286.7)	.69
While blood cell (/μL)	5000‐12000	8575 (2780‐16490)	3995 (2200‐12310)	2720 (1360‐5740)	5120 (1330‐15210)	.71
Neutrophils (/μL)	2180‐6960	4104 (670‐11496)	1938 (880‐9250)	1293 (510‐2813)	2359 (290‐14300)	.71
Band neutrophils (/μL)	0	0 (0‐1223)	0 (0‐800)	0 (0‐231)	0 (0‐334)	.48
Lymphocytes (/μL)	1320‐5860	2573 (930‐6554)	1292 (120‐2610)	1045 (710‐2500)	1630 (670‐3890)	.68
Eosinophils (/μL)	10‐1000	0 (0‐113)	0 (0‐984)	0 (0‐114)	0 (0‐330)	.31
Basophils (/μL)	0‐1200	0 (0‐113)	0 (0‐369)	0 (0‐70)	0 (0‐190)	.51
Platelets (/μL)	90000‐360000	99808 ± 34310	121900 ± 42969	106313 ± 44846	96113 ± 38195	.16
Fibrinogen (mg/dL)	150‐375	640 (399‐1116)	549 (335‐1396)	444 (331‐777)	417 (109‐826)	.59
Glucose (mg/dL)	72‐114	131.5 (105.4‐353.3)	128 (50.8‐173)	124.8 (91.9‐180.4)	136.4 (80.4‐286.7)	.69
Creatinine (mg/dL)	0.6‐1.8	2.2 (1.2‐6.4)	1.6 (1.2‐5.6)	1.3 (0.9‐2.7)	1.3 (0.7‐5.8)	.54
Sodium (mmol/L)	132‐141	125.96 ± 5.51^*^	131.13 ± 4.64	131 ± 2.85	130.86 ± 6.02	.002
Potassium (mmol/L)	2.70‐4.90	3.36 (2.12‐5.48)	3.38 (2.75‐5.37)	3.25 (2.52‐4.14)	3.51 (2.31‐7.4)	.48
Chloride (mmol/L)	94‐102	92 (70‐102)	95 (84‐101)	98 (88‐102)	94 (75‐107)	.74
tCO2 (mmol/L)	24‐31	22 (12.5‐33.1)	26.9 (12.5‐31.5)	27.15 (24.2‐31.2)	27.45 (22.2‐35.4)	.73
Total calcium (mg/dL)	10.7‐13.4	9.58 (6.07‐11.26)	10.34 (6.91‐11.32)	10.64 (10.27‐11 39)	10.77 (7.73‐12.26)	.63
Phosphorus (mg/dL)	1.9‐5.4	5.54 (3.1‐15.29)	3.89 (1.39‐6.49)	2.9 (0.89‐4.78)	3.51 (1.91‐9.18)	.63
Total protein (g/dL)	4.6‐6.9	6.65 ± 1.39	6.48 ± 0.78	6.64 ± 0.52	6.48 ± 1.13	.93
Albumin (g/dL)	2.5‐4.2	2.66 ± 0.59	2.61 ± 0.37	2.88 ± 0.23	2.96 ± 0.60	.08
AST (U/L)	205‐555	350 (202‐1093)	539.5 (164‐1208)	310 (223‐513)	350.5 (193‐615)	.63
CK (U/L)	90‐270	223 (118‐11472)	490.5 (110‐13091)	186 (86‐433)	182 (58‐1536)	.68
GGT (U/L)	12‐45	29 (13‐67)	29 (12‐386)	28.5 (23‐48)	28 (18‐63)	.51
Total bilirubin (mg/dL)	0.1‐1.9	3.6 (1.5‐10.2)	4.45 (1.9‐12.3)	3.65 (1.7‐7.4)	3.6 (1.5‐16)	.67

## 4 DEVELOPMENT OF LAMINITIS

Seventeen of 85 (20%) horses developed laminitis. Laminitis was diagnosed in 14/17 horses based on a combination of clinical signs and radiographic findings. Radiographic findings included rotation of the distal phalanx relative to the dorsal hoof wall (range, 2‐12°) in 4 horses, sinking of the distal phalanx within the hoof capsule with no rotation in 3 horses, and a combination of rotation (range, 2‐7°) and sinking of the distal phalanx in 7 horses. Laminitis was diagnosed solely on clinical signs (severe lameness, Obel grade 4, increased digital pulse pressure, clefting of the coronary band) in 3 horses. These 3 horses were presented with signs of laminitis at time of admission and had a final diagnosis of neorickettsiosis. Laminitis was confirmed at necropsy for these 3 horses. Laminitis stratified by final diagnosis is shown in Table 1. Neorickettsiosis cases (11/26 [42%]) were significantly more likely to develop laminitis than coronavirus cases (0/16 [0%]; odds ratio [OR] 24.48; 95% confidence interval [CI]: 1.33‐451.73), *P* = .03), but salmonellosis cases (3/20 [15%]) and unknown (3/23 [13%]) were not more likely to develop laminitis than coronavirus cases (OR 6.6; 95% CI: 0.31‐137.78; *P* = .22 and OR 5.63; 95% CI: 0.27‐116.99; *P* = .26, respectively). When compared to neorickettsiosis, unknown (OR 0.23; 95% CI: 0.06‐0.9; *P* = .04) and coronavirus (OR 0.04; 95% CI: 0.002‐0.75; *P* = .03) cases were less likely to develop laminitis, but there was no difference between salmonellosis and neorickettsiosis cases (OR 0.27; 95% CI: 0.07‐1.07; *P* = .06). Supplemental Table 1 summarizes univariate logistic regression results for the prediction of laminitis. The final multivariable logistic model for prediction of laminitis included admission heart rate, admission total solids, band neutrophils, and bicarbonate concentration (Table 3). The final predictive model had excellent agreement with an area under the curve of 0.95. Sensitivity and specificity for the final predictive model were 86% and 84%, respectively.

**TABLE 3:** Multivariable logistic regression model for prediction of laminitis in 85 horses hospitalized for acute colitis

Variable	Odds ratio (95% CI)	*P* value
Admission heart rate (bpm)	1.08 (1.02‐1.15)	.01
Total solids (g/dL)	0.17 (0.06‐0.54)	.003
Band neutrophils (/μL)	1248.47 (6.62‐235 540.1)	.008
tCO_2_ (mmol/L)	0.68 (0.50‐0.92)	.01

### 4.1 Survival

Sixty‐three of 85 (74.1%) horses survived to discharge. All (16/16, 100%) coronavirus cases survived to discharge. Seventeen of 26 (65.4%) neorickettsiosis cases survived to discharge, 14/20 (70%) salmonellosis cases survived to discharge, and 16/23 (69.6%) unknown cases survived to discharge. Of 22 nonsurvivors, 20 were euthanized and 2 died (1 salmonellosis, 1 unknown) during hospitalization. Of 22 nonsurvivors, 13 had laminitis, and 9 did not have laminitis. Because of 100% survival in the coronavirus cases, survival analysis resulted in exceptionally high hazard ratios (HRs) or incalculable *P* values, depending on the referent. When compared to neorickettsiosis cases, coronavirus cases were less likely to die (HR: 1.41 × 10^−20^; 95% CI: incalculable, *P* = incalculable), whereas there was no difference for salmonellosis (HR: 0.49, 95% CI: −0.03 to 1.01, *P* = .07) or unknown cases (HR: 0.79, 95% CI: 0.006‐1.57, *P* = .05). Supplemental Table 2 summarizes univariate Cox regression results for the prediction of survival. The final multivariate Cox regression model for prediction of nonsurvival included admission packed cell volume, admission L‐lactate concentration, and the development of laminitis (Table 4).

**TABLE 4:** Multivariable Cox regression model for prediction of nonsurvival in 85 horses hospitalized for acute colitis

Variable	Hazard ratio (95% CI)	*P* value
Packed cell volume (%)	1.17 (1.09‐1.26)	<.001
L‐lactate (mmol/L)	1.33 (1.14‐1.55)	<.001
Laminitis ‐ yes	7.07 (1.67‐29.95)	.008

## 5 DISCUSSION

Our retrospective study reports and compares the clinical and clinicopathologic features of acute colitis because of enteric salmonellosis, enteric coronavirus, and enteric neorickettsiosis in a population of hospitalized horses. There were few differences in the clinical features across diagnoses. However, horses with enteric coronavirus were less likely to develop laminitis than horses with neorickettsiosis. All horses (16/16) with enteric coronavirus survived to discharge, whereas 17/26 horses with neorickettsiosis survived to discharge; however, complete separation of data complicated statistical comparison of survival between groups.

Coronavirus cases were less likely to have diarrhea and more likely to display colic signs during hospitalization, and this supports the clinical impression within our hospital. However, with the exception of sodium concentration as discussed below, there were no significant differences for clinicopathologic findings between groups. This supports the findings of a previous study which determined that the disease features of enteric coronavirus and salmonellosis were similar in horses and supports the difficulty in distinguishing etiologic agents of colitis based on clinical findings.^5^ Of note, our record search did identify coinfection with coronavirus and *Salmonella* in a single horse, although this horse was excluded for the final study. Coinfection with coronavirus and *Salmonella* has been reported in a horse.^2^

In contrast to a previous study of enteric coronavirus and salmonellosis, where a single horse developed laminitis, 20% of horses in our study developed laminitis.^5^ The incidence of laminitis associated with colitis in the veterinary literature varies, from a low of 2.4% in the aforementioned study of salmonellosis and coronavirus cases, to 36% in a study of neorickettsiosis cases.^5^, ^8^ Almost two‐thirds (11/17) of laminitis cases in our study were associated with neorickettsiosis, whereas the remainder were salmonellosis and unknown cases. The prevalence of neorickettsiosis in our geographical region likely explains the difference in laminitis rates as compared to the previous study, as the region of the country in which that study was performed is not affected by enteric neorickettsiosis.^5^ No coronavirus cases in our study developed laminitis, and this coincides with both our clinical impression and prior research.^2^, ^4^, ^5^

Digital cryotherapy in horses with colitis reduces the incidence of laminitis from 33% in horses not treated with cryotherapy to 10% in horses treated with cryotherapy.^10^ Importantly, cryotherapy has been most effective at prevention of laminitis, not necessarily treatment of laminitis. Despite the more widespread use of digital cryotherapy in colitis cases as a preventative method against laminitis, regions of the country continue to see higher incidence of laminitis, most commonly associated with neorickettsiosis. In fact, the study evaluating digital cryotherapy found that 7 horses developed laminitis despite digital cryotherapy, and 6 of those 7 were neorickettsiosis cases.^10^ In our study, neorickettsiosis cases were significantly more likely to present with or to develop laminitis than coronavirus and unknown cases, despite the widespread use of digital cryotherapy in colitis cases in our hospital. Recent literature has compared various methods for digital cryotherapy, and while 1 study found that the commercially available sleeve‐style ice boot used in our hospital achieved appropriately low skin and lamellar temperatures,^13^ another study found that this sleeve‐style boot did not maintain hoof surface temperatures below 10°C.^14^ It is possible that the combination of variably effective cryotherapy method, warm ambient temperature seen in summer when neorickettsiosis cases are most common, as well as the fact that some cases presented to the hospital already showing signs of laminitis, contributed to the high incidence of laminitis in our study. Beyond the study evaluating digital cryotherapy in colitis cases,^10^ the majority of studies evaluating methods of digital cryotherapy are performed in ideal research settings, and one can question how these results translate to clinical practice.

Despite neorickettsiosis being a significant predictor of laminitis in the univariate analysis, this relationship did not hold in the multivariable analysis. The final model for laminitis prediction in our study included admission heart rate, admission total solids, band neutrophils, and bicarbonate concentration. The elevated heart rate and presence of immature neutrophils at admission could indicate more severe disease or could suggest systemic inflammatory response syndrome in the horses in this study.^15^ A previous study of systemic inflammatory response syndrome (SIRS) in 105 adult horses admitted on emergency found that detection of band neutrophils at admission was associated with SIRS, as well as with a poor outcome.^16^ Lower total solids and bicarbonate concentrations were also predictive of a higher risk of laminitis in our study, and this likely reflects that horses with hypoproteinemia and acidosis were more severely ill. Previous research evaluating risk factors for laminitis in hospitalized horses identified high PCV, low total solids, diarrhea, endotoxemia, and pneumonia as risk factors for development of laminitis in hospitalized horses.^7^

Horses who developed laminitis were less likely to survive, and as the development of laminitis was retained in the final model for prediction of nonsurvival, development of laminitis is likely associated with the lower survival rate noted in enteric neorickettsiosis cases, although this could be confounded by the fact that a poor prognosis associated with severe laminitis might have led to an owner's decision to euthanize their horse. The majority (59%) of nonsurvivors had laminitis and there is likely confounding present in the finding that laminitis is a predictor of nonsurvival.

Our study identified a higher nonsurvival rate than a previous study of enteric coronavirus and salmonellosis in 42 horses (25.9% vs 2.4% nonsurvival).^5^ Nonsurvival rates in studies of acute colitis in horses have varied from 2.4% to as high as 43%.^5^, ^17^, ^18^ In our study, neorickettsiosis cases appeared less likely to survive than coronavirus cases, but not different from salmonellosis or unknown cases, although complete separation of data complicated statistical comparison of survival between groups. Neorickettsiosis cases had lower sodium concentration than the other groups, which might be because of profuse diarrhea and gastrointestinal loss of sodium in more severely diarrheic cases. Because of the retrospective nature of the study, information regarding duration and severity of clinical signs before referral is not available. It is possible that the higher nonsurvival rate in this study is related to selection bias of the referring veterinarian as to which cases were presented to the referral hospital versus which cases were treated in the field. Some horses presented already having signs of laminitis, suggesting that more severe cases were referred in to our hospital. Additionally, we have recognized differences in severity of neorickettsiosis from year to year, and it is possible that the years enrolled in our study were particularly severely affected years.

The final model for prediction of nonsurvival included packed cell volume and L‐lactate concentration at admission as well as the development of laminitis. It is likely that more severely affected cases had more severe hemoconcentration and compromised perfusion. Because many of the horses who developed laminitis were euthanized because of the poor prognosis and poor quality of life associated with the severity of their laminitis, it is not surprising that laminitis was retained in the final model for nonsurvival.

Limitations of our study include the small number of animals per group and the retrospective nature of the study. Although significant differences between neorickettsiosis and salmonellosis cases for development of laminitis and nonsurvival were not found in our study, it is likely with a larger population size these results could have reached statistical significance. Additionally, it is possible that the unknown group might have included false negative neorickettsiosis, coronavirus or salmonellosis, or coronavirus and salmonellosis, cases, and this might have reduced our statistical power. A recent study suggested that a 2‐step approach of fecal and nasal swab testing should be utilized for the diagnosis of coronavirus in horses.^19^ This approach was not used in any of the horses in this study because of the retrospective nature of the study, but could be indicated in the future for coronavirus testing in horses. We initially performed statistical analysis both with and without inclusion of the unknown group, and its presence or exclusion did not lead to significant differences in results. Furthermore, survival analysis was complicated by the lack of death in the coronavirus group, resulting in astronomically high HRs or incalculable *P* values when comparing survival between groups. Finally, as previously mentioned, the development of severe laminitis might have led to the decision to euthanize a horse and therefore its inclusion in the final model for nonsurvival could be misleading.

In conclusion, our study shows that horses with enteric neorickettsiosis are more likely to develop laminitis than horses with coronavirus. Additionally, we identified risk factors for the development of laminitis and for nonsurvival. This information can be applied in the future to predict the risk of laminitis and nonsurvival in a clinical setting. Further research with multi‐institutional studies is indicated to determine national rates of laminitis and survival in equine colitis cases.

## CONFLICT OF INTEREST DECLARATION

Authors declare no conflict of interest.

## OFF‐LABEL ANTIMICROBIAL DECLARATION

Authors declare no off‐label use of antimicrobials.

## INSTITUTIONAL ANIMAL CARE AND USE COMMITTEE (IACUC) OR OTHER APPROVAL DECLARATION

Authors declare no IACUC or other approval was needed.

## HUMAN ETHICS APPROVAL DECLARATION

Authors declare human ethics approval was not needed for this study.

## Supporting information


**Supplemental Table 1** Univariate logistic regression for prediction of laminitis in 85 horses hospitalized for acute colitis.

**Supplemental Table 2** Univariate Cox regression for prediction of non‐survival in 85 horses hospitalized for acute colitis.
