# Supplementary material for: Risk factors for laminitis and nonsurvival in acute colitis: Retrospective study of 85 hospitalized horses (2011‐2019)
Source: J Vet Intern Med. 2021 May 3;35(4):2019–25. doi: 10.1111/jvim.16147 (PMC8295695; doi:10.1111/jvim.16147)
Supplement: Supplementary file 1 — Supplemental Table 1 Univariate logistic regression for prediction of laminitis in 85 horses hospitalized for acute colitis. [file JVIM-35-2019-s001.pdf]

Supplemental Table 1 - Univariate logistic regression for prediction of laminitis in 85 horses hospitalized for acute colitis.

Variable	Odds ratio (95% CI)	P value
**Final diagnosis**		
**Coronavirus**	*Referent*	
**Neorickettsiosis**	24.48 (1.33 - 451.73)	0.03
**Salmonellosis**	6.6 (0.32 - 137.78)	0.22
**Unknown**	5.63 (0.27 - 116.99)	0.26
**Admission temperature (degF)**	1.33 (0.96 - 1.84)	0.09
**Admission heart rate (bpm)**	1.06 (1.02 - 1.1)	0.003
**Packed cell volume (%)**	1.02 (0.97 - 1.08)	0.37
**Total solids (g/dL)**	0.47 (0.28 - 0.79)	0.004
**Lactate (mmol/L)**	1.15 (0.95 - 1.39)	0.14
**Blood glucose (mg/dL)**	0.99 (0.98 - 1.01)	0.71
**White blood cells (/µL)**	1.17 (1.02 - 1.33)	0.02
**Neutrophils (/µL)**	1.11 (0.95 - 1.3)	0.19
**Band neutrophils (/µL)**	27.31 (2.24 - 332.64)	0.01
**Lymphocytes (/µL)**	1.67 (1.13 - 2.48)	0.01
**Eosinophils (/µL)**	0.03 (1.09e-06 - 989.8)	0.52
**Basophils (/µL)**	0.009 (1.01e-09 - 91707.29)	0.57
**Platelet count (/µL)**	1.003 (0.99 - 1.02)	0.67
**Fibrinogen (mg/dL)**	1.002 (0.99 - 1.004)	0.23
**Glucose (mg/dL)**	0.996 (0.98 - 1.01)	0.61
**Creatinine (mg/dL)**	1.11 (0.72 - 1.71)	0.64
**Sodium (mmol/L)**	0.98 (0.89 - 1.08)	0.66
**Potassium (mmol/L)**	0.9 (0.47 - 1.72)	0.76
**Chloride (mmol/L)**	1.01 (0.94 - 1.1)	0.73
**tCO_2 (mmol/L)**	0.93 (0.84 - 1.03)	0.14
**Total calcium (mg/dL)**	0.74 (0.5 - 1.09)	0.13
**Phosphorus (mg/dL)**	1.21 (0.96 - 1.52)	0.11
**Total protein (g/dL)**	0.42 (0.22 - 0.78)	0.006
**Albumin (g/dL)**	0.13 (0.03 - 0.51)	0.003
**AST (U/L)**	1.00 (0.99 - 1.002)	0.92
**CK (U/L)**	1.00 (0.99 - 1.00)	0.5
**GGT (U/L)**	0.98 (0.933 - 1.03)	0.39
**Total bilirubin (mg/dL)**	0.91 (0.69 - 1.18)	0.47
