# Supplementary material for: Risk factors for laminitis and nonsurvival in acute colitis: Retrospective study of 85 hospitalized horses (2011‐2019)
Source: J Vet Intern Med. 2021 May 3;35(4):2019–25. doi: 10.1111/jvim.16147 (PMC8295695; doi:10.1111/jvim.16147)
Supplement: Supplementary file 2 — Supplemental Table 2 Univariate Cox regression for prediction of non‐survival in 85 horses hospitalized for acute colitis. [file JVIM-35-2019-s002.pdf]

Supplemental Table 2 – Univariate Cox regression for prediction of non-survival in 85 horses hospitalized for acute colitis.

Variable	Hazard ratio (95% CI)	P value
**Final diagnosis**		
**Coronavirus**	1.41 x10^{-20} (incalculable)	Incalculable
**Neorickettsiosis**	Referent	*
**Salmonellosis**	0.49 (-0.03 - 1.01)	0.07
**Unknown**	0.79 (0.01 - 1.57)	0.05
**Laminitis – yes**	4.51 (1.89 - 10.78)	0.0008
**Admission temperature (degF)**	0.98 (0.76 - 1.26)	0.86
**Admission heart rate (bpm)**	1.05 (1.03 - 1.08)	<0.001
**Packed cell volume (%)**	1.07 (1.04 - 1.11)	<0.001
**Total solids (g/dL)**	0.72 (0.49 - 1.06)	0.09
**Lactate (mmol/L)**	1.29 (1.14 - 1.45)	<0.001
**Blood glucose (mg/dL)**	1.01 (1.01 - 1.02)	0.002
**White blood cells (/µL)**	1.19 (1.08 - 1.32)	<0.001
**Neutrophils (/µL)**	1.15 (1.03 - 1.29)	0.01
**Band neutrophils (/µL)**	2.76 (0.95 - 7.99)	0.06
**Lymphocytes (/µL)**	1.55 (1.2 - 1.99)	0.001
**Eosinophils (/µL)**	0.03 (5.23e-06 - 190.8)	0.44
**Basophils (/µL)**	0.04 (2.19e-06 - 869.91)	0.54
**Platelet count (/µL)**	0.99 (0.99 - 1.01)	0.34
**Fibrinogen (mg/dL)**	1.00 (0.99 - 1.004)	0.17
**Glucose (mg/dL)**	1.02 (1.01 - 1.03)	<0.001
**Creatinine (mg/dL)**	1.47 (1.12 - 1.93)	0.005
**Sodium (mmol/L)**	0.97 (0.9 - 1.05)	0.47
**Potassium (mmol/L)**	1.5 (1.04 - 2.14)	0.03
**Chloride (mmol/L)**	0.96 (0.9 - 1.02)	0.21
**tCO_2 (mmol/L)**	0.92 (0.85 - 0.99)	0.03
**Total calcium (mg/dL)**	0.64 (0.48 - 087)	0.004
**Phosphorus (mg/dL)**	1.3 (1.09 - 1.54)	0.003
**Total protein (g/dL)**	0.69 (0.45 - 1.08)	0.1
**Albumin (g/dL)**	0.61 (0.24 - 1.55)	0.3
**AST (U/L)**	1.00 (0.99 - 1.002)	0.35
**CK (U/L)**	1.00 (0.99 - 1.000)	0.1
**GGT (U/L)**	1.0 (0.99 - 1.007)	0.79
**Total bilirubin (mg/dL)**	1.05 (0.89 - 1.25)	0.57
